# Modulating TRPV1 and TRPA1 channels: a viable strategy for treating asthma using Chinese herbal medicines

**DOI:** 10.3389/fphar.2025.1573901

**Published:** 2025-07-03

**Authors:** Xiang Yao, Xuejian Zhang, Tao Cui, Meiling Jian, Hao Wu, Chunjie Wu, Feiyan Tao

**Affiliations:** ^1^ Sichuan Sanlian New Material Co., Ltd., Chengdu, China; ^2^ State Key Laboratory of Southwestern Chinese Medicine Resources, School of Pharmacy, Chengdu University of Traditional Chinese Medicine, Chengdu, China; ^3^ Research and Development Centre, China Tobacco Sichuan Industrial Co. Ltd., Chengdu, China; ^4^ Innovative Institute of Chinese Medicine and Pharmacy/Academy for Interdiscipline, Chengdu University of Traditional Chinese Medicine, Chengdu, Sichuan, China; ^5^ Sichuan Engineering Research Center for Endangered Medicinal Animals, Chengdu, Sichuan, China

**Keywords:** asthma, alleviation, TRPV1/TRPA1 channels, modulation, Chinese herbal medicines

## Abstract

Asthma represents a significant global health challenge, imposing a substantial burden on society worldwide. Over the past decades, the development of asthma medications has significantly reduced asthma-related mortality. However, current pharmacological treatment regimens have not yet demonstrated the capacity to induce a durable remission of asthma. Transient receptor potential channels, specifically vanilloid receptor 1 (TRPV1) and ankyrin 1 (TRPA1), are polymodal sensory channels extensively distributed in the lungs and represent promising therapeutic targets for asthma. Increasing evidence suggests that Chinese herbal medicines (CHM) exert beneficial effects in asthma treatment by modulating TRPV1/TRPA1. Therefore, this study systematically analyzed 134 articles covering the pathogenesis of asthma, current treatment strategies, the role of TRPV1/TRPA1 in asthma, and the modulation of TRPV1/TRPA1 by Chinese herbal medicines in asthma. In summary, our review comprehensively elaborates on how CHM modulates TRPV1/TRPA1 channels to alleviate pulmonary inflammation. These findings provide viable options for asthma drug discovery and establish the foundation for developing effective CHM-based therapeutics.

## Graphical Abstract

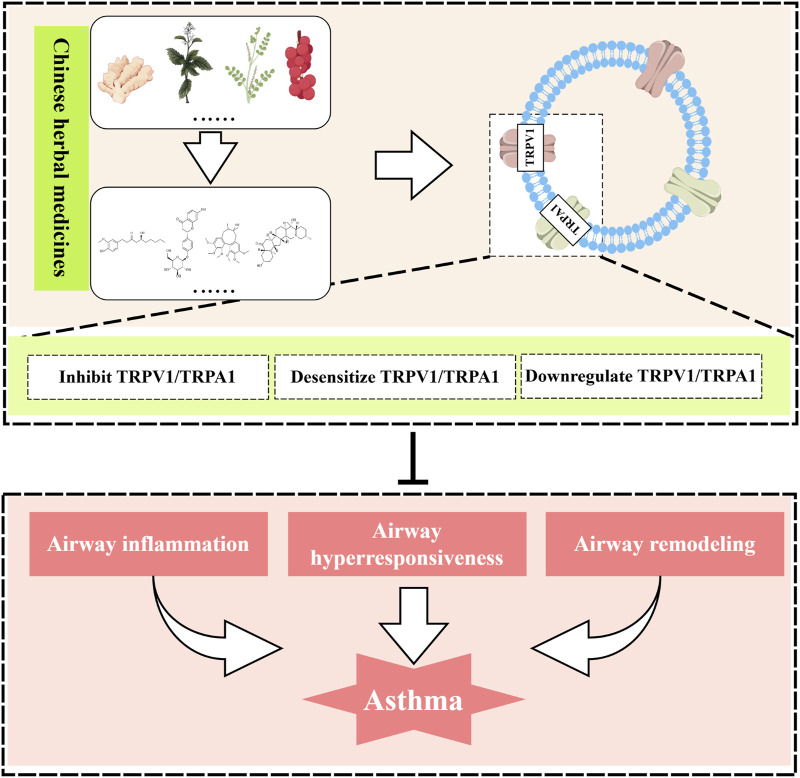

## Introduction

Asthma is a prevalent chronic inflammatory disease characterized by increased mucus secretion in the airways, obstruction, airway hyperresponsiveness, and airway remodeling, presenting with a spectrum of typical clinical symptoms, including cough, chest tightness, wheezing, and dyspnea (Porsbjerg et al., 2023). Asthma constitutes a significant global burden, leading directly to substantial healthcare expenditures and affecting patients’ learning, work, and quality of life profoundly (Yaghoubi et al., 2019). As of 2019, there were a total of 262 million asthma sufferers across 204 countries and regions globally, with a prevalence rate of 4.2% among individuals aged 20 and above in China, amounting to approximately 45.7 million people (Huang et al., 2019; Shin et al., 2023). With the advancement of modern medicine, drugs such as corticosteroids, β2-adrenergic agonists, and certain biologics have been used to treat asthma, demonstrating positive effects. Nevertheless, the long-term use of corticosteroids carries risks of systemic side effects such as osteoporosis and metabolic disturbances, while *β*_2_-agonists can induce tachyphylaxis and cardiovascular complications. Moreover, biological agents exhibit variable efficacy among asthma endotypes and remain cost-prohibitive for global implementation (Lommatzsch et al., 2024). Therefore, the development of asthma medications is essential to achieve both the short-term goal of supplementing or replacing existing treatments and the long-term goal of curing asthma.

The pathogenesis of asthma is complex, with inflammation playing a crucial role. Asthma is characterized primarily by chronic inflammation of the lungs, involving various immune cells, cytokines, and neurotransmitters related to the immune and nervous systems, all of which collectively regulate the progression of inflammation (Boonpiyathad et al., 2019; Zhang et al., 2022). TRP channels are a class of non-selective ion channels that can detect various physical and chemical signals both inside and outside the body, serving as the body’s “signal detectors.” TRPV1 and TRPA1 channels, widely expressed in both neural and non-neural compartments of pulmonary tissue, serve as critical regulators of airway physiology and pathology (Zholos, 2015). Emerging evidence indicates that these channels not only mediate sensory nerve signaling but also act as key modulators of inflammatory cascades (Diver et al., 2022). Under physiological conditions, balanced activation of TRPV1 and TRPA1 contributes to airway homeostasis through mechanisms such as mucociliary clearance, pathogen defense, and immunomodulation. Conversely, dysregulation of these channels has been implicated in the pathogenesis of respiratory diseases, particularly asthma, by driving core pathological features, including chronic inflammation and airway remodeling. Mechanistically, TRPV1/TRPA1 activation potentiates neurogenic inflammation by inducing the release of sensory nerve-derived neuropeptides. These neuropeptides directly trigger mast cell degranulation and leukocyte recruitment (Undem and Taylor-Clark, 2014). Concurrently, these channels modulate immune cell dynamics by promoting Th2 polarization, dendritic cell maturation, and eosinophil infiltration, thereby amplifying immune-driven inflammation (Basu and Srivastava, 2005; Bertin et al., 2014). This dual neuro-immune inflammatory axis synergistically exacerbates airway hyperresponsiveness, mucus hypersecretion, and structural remodeling. Given their pivotal roles in asthma pathophysiology, TRPV1 and TRPA1 have emerged as promising therapeutic targets for developing novel disease-modifying agents. To date, numerous studies have demonstrated that the multi-metabolite and multi-target characteristics of CHM can alleviate airway inflammation through various pathways, showing considerable advantages in relieving asthma symptoms (Li et al., 2015; Zhou et al., 2022).

In this paper, we provide an overview of the ameliorative or therapeutic effects of CHM or their extracts on asthma, offering a reference for the development of new drugs modulating TRPV1/TRPA1 for asthma treatment.

## Review methodology

To investigate the potential mechanisms by which CHM treats asthma through modulation of TRPV1/TRPA1, we systematically searched relevant literature in the PubMed, Web of Science, ScienceDirect, and CNKI databases. The search strategy employed key terms including “Asthma, pathogenesis, therapeutic strategies,” “Asthma, TRPV1/TRPA1,” and “Asthma, Chinese herbal Medicine/Chinese herbal Medicine metabolites.” Based on the systematically retrieved results, we first reviewed the pathogenesis of asthma and the limitations of current therapeutic strategies, with a specific focus on delineating the roles of TRPV1 and TRPA1 in the asthmatic disease process. This was followed by an in-depth analysis and discussion of the research evidence concerning CHM-mediated regulation of TRPV1 or TRPA1 for asthma treatment. Literature screening was rigorously performed by two independent reviewers according to predefined criteria. Inclusion criteria encompassed articles elucidating the specific mechanisms of TRPV1 or TRPA1 in regulating asthma, as well as studies exploring the mechanistic basis of TCM’s anti-asthmatic effects through TRPV1/TRPA1 modulation. Exclusion criteria covered grey literature, editorials, and duplicate publications.

## Asthma

### Pathogenesis of asthma

**FIGURE 1:**
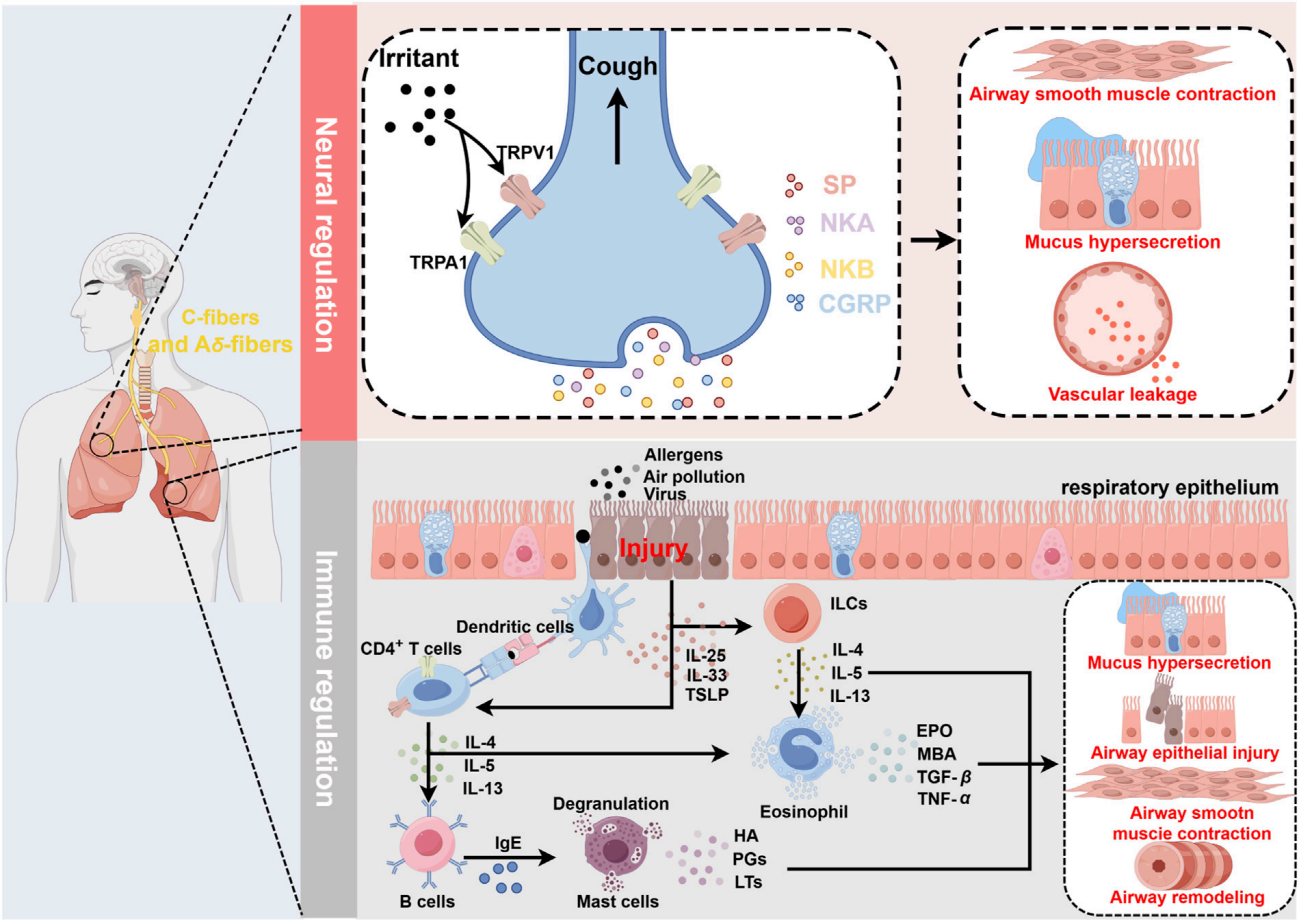
Schematic diagram of asthma pathogenesis. Neural regulation: Irritants act on TRPV1/TRPA1 receptors located on sensory C fibers and A*δ* fibers, inducing a cough reflex and promoting the release of SP, NKA, NKB, and CGRP from nerve endings. This leads to airway smooth muscle contraction, increased mucus secretion, and vasodilation, resulting in plasma protein and inflammatory cell extravasation, all of which contribute to the symptoms during the acute phase of asthma. Immune regulation: Airways epithelial damage increases the exposure to allergens or other irritants. DCs recognize allergens and present them to naïve T cells, which are then induced to differentiate into Th2 cells and secrete IL-4, IL-13, and IL-5. Additionally, damaged epithelial cells secrete IL-25, IL-33, and TSLP, which activate ILC2s, leading to the secretion of IL-4, IL-13, and IL-5. These inflammatory mediators activate eosinophils, which release EPO, MBP, TGF-β, and TNF-α. Upon antigen recognition, mast cells undergo degranulation, releasing HA, PGs, and LTs. The interactions among these immune cells and their secreted inflammatory mediators further exacerbate airway epithelial damage, leading to increased airway hyperresponsiveness and airway remodeling, thereby promoting the worsening of asthma symptoms. In this process, TRPV1/TRPA1 can promote the differentiation and maturation of multiple immune cells, including T cells and DCs, thus participating in the regulation of immune-mediated inflammation in asthma. Created with figdraw.com.

Asthma is a heterogeneous condition influenced by a multifaceted array of factors, including genetic and environmental components. To facilitate diagnosis and enable personalized asthma treatment, researchers have classified asthma based on different clinicopathological features, triggers, severity, molecular biomarkers, or other factors. In recent years, a classification of asthma into high Th2 level and low Th2 level based on Th2 inflammatory factors has been widely accepted (Kuruvilla et al., 2019). Here, the main pathological processes of asthma are briefly outlined (Figure 1).

Airway epithelial cells, tightly arrayed across the entire airway surface, constitute the body’s first line of defense. In the early stages of asthma, allergens and air pollutants compromise the integrity of the airway epithelial barrier, triggering neurogenic inflammation and immune responses. Damage to the airway epithelium leads to the exposure of sensory nerve endings, and allergen stimulation induces depolarizing currents in these nerve endings, triggering the cough reflex and the release of neuropeptides such as substance P (SP), neurokinin A (NKA), neurokinin B (NKB), and calcitonin gene-related peptide (CGRP). These neuropeptides cause airway congestion, edema, increased mucus secretion, and bronchial smooth muscle contraction, exacerbating acute asthma attacks (Undem and Taylor-Clark, 2014). Furthermore, airway damage increases epithelial permeability, enhancing allergen exposure and promoting the release of alarmins [Interleukin-25 (IL-25), Interleukin-33 (IL-33), and thymic stromal lymphopoietin (TSLP)] by epithelial cells, which in turn induce Th2-high asthma (Gaspar et al., 2020; Gao and Rezaee, 2022). Type 2 innate lymphoid cells (ILC2s) are primarily located in mucosal tissues, where they sense and respond to relevant cytokines and signals, acting as key initiators of type 2 immune responses. Due to their lack of antigen-specific receptors, ILC2s primarily trigger non-specific immune responses. Epithelial-derived cytokines IL-33 and IL-25 rapidly activate ILC2s, leading to the release of type 2 cytokines, including IL-4, IL-13, and IL-5 (Maggi et al., 2021). Dendritic cells (DCs) play a crucial role in initiating and amplifying type 2 immune responses. During the immune response, DCs capture allergens that penetrate through damaged epithelial barriers and process them into small peptides. Epithelial-derived TSLP enhances the expression of surface MHC-II and costimulatory molecules on DCs, promoting their differentiation and maturation. Mature DCs rapidly migrate to draining lymph nodes (LNs), where they present processed MHC peptide complexes to naive T cells, inducing their differentiation into Th2 cells (Morianos and Semitekolou, 2020). Upon re-exposure to allergens, Th2 cells are quickly activated, secreting type 2 inflammatory cytokines such as IL-4, IL-13, and IL-5, thereby promoting B cell synthesis of immunoglobulin E (IgE). IgE binds to the Fc*ε*RI receptors on the surface of mast cells (MCs), triggering their degranulation and the release of pro-inflammatory mediators such as histamines, prostaglandins, and leukotrienes, which result in airway smooth muscle contraction, bronchospasm, altered vascular permeability, and increased mucus secretion (Galli et al., 2020). Concurrently, IL-4 and IL-13 disrupt the barrier proteins of airway epithelial cells, exacerbating barrier dysfunction (Saatian et al., 2013). Increasing evidence indicates that eosinophils play a crucial role in the pathophysiology of asthma. Eosinophilia has been identified as an essential marker of Th2-high asthma. Eosinophils are recruited to the lungs in response to chemokines secreted by damaged epithelial cells and are subsequently activated by IL-5, which is secreted by lung Th2 cells. They release granule proteins, such as eosinophil peroxidase (EPO), major basic protein (MBP), transforming growth factor-β (TGF-β), and tumor necrosis factor-α (TNF-α), which alter the activation thresholds of sensory and parasympathetic nerves and are involved in the persistent airway epithelial cell damage and repair process. This leads to airway hyper-responsiveness and airway remodeling, potentially having profound impacts on airway function and directly worsening asthma symptoms (Drake et al., 2018).

Due to the lack of specific biomarkers, researchers define asthma with the absence of Th2 biomarkers (such as increased blood and sputum eosinophils and elevated FeNO) as Th2-low asthma. However, the pathophysiological mechanisms of Th2-low asthma are not yet well understood. Over the years, immune responses mediated by neutrophils (involving Th1 and Th17 cells) have emerged as a potential mechanism for Th2-low asthma. However, this is controversial due to the failure of attempts targeting these immune pathways (Nair et al., 2021). Additionally, it was found that the onset of asthma could also be independent of immune responses or inflammation (Habib et al., 2022). Further discussion would not be provided due to the limited studies in this area.

### Present treatment strategy for asthma

Asthma has long been regarded as a difficult-to-cure disease due to its extremely complex pathological mechanisms and clinical manifestations. Currently, there is a consensus among experts that the aims of asthma treatment mainly focus on relieving asthma symptoms, reducing the frequency of asthma attacks, and reducing the burden of treatment on patients rather than curing asthma patients (Menzies-Gow et al., 2020). Increasing drugs and techniques have been applied to the prevention and treatment of asthma, mainly including conventional drug therapy such as corticosteroids, *β*_2_-agonists, cholinergic receptor blockers, immunotherapy such as new biologically targeted agents, and surgical treatment. Appropriate therapeutic measures should be selected for different asthma phenotypes of patients, which will effectively alleviate the disease and improve their quality of life.

It is a well-established fact that the development of asthma is primarily caused by inflammation (Busse, 1998). With the application of inhaled corticosteroids (ICS), the treatment of asthma has undergone a fundamental change. It is widely used in mild and moderate asthma patients, and its long-term use plays an important role in relieving inflammatory symptoms, preventing disease exacerbation, and alleviating pulmonary dysfunction. Nowadays, ICS has been proven to reduce airway wall area and thickness in clinical research, thereby becoming the foundation of current asthma treatment (Hoshino et al., 2016). Asthma is often associated with airway hyper-responsiveness, and long-acting *β*_2_-agonists (LABA) could act on airway smooth muscle to rapidly alleviate asthma symptoms. It is important to note that *β*_2_-agonists provide only relief of asthma symptoms; the underlying disease may be active while the patient is in symptomatic remission. Historical tragedies have shown that targeting symptoms alone without relieving inflammation can be fatal. According to the latest Global Initiative for Asthma (GINA) recommendations, short-acting *β*_2_-agonists should not be used alone, for prolonged periods of time, or in excessive doses (Krings and Beasley, 2024). The combination of LABA and ICS has been shown to be more effective in the control of asthma (Cardet et al., 2023). Anticholinergics have been shown to reduce airway mucus secretion and airway hyper-responsiveness along with bronchiolar dilation. They are often used in combination with ICS and LABA in refractory asthma to reduce hormone dosage and improve lung function (Kim et al., 2021). Leukotriene receptor antagonists act as anti-inflammatory and bronchodilator agents, and theophyllines function as smooth muscle relaxants and respiratory stimulants, and both serve as adjunctive therapies. Over the past decade, personalized therapeutic strategies for asthma have become increasingly mainstream, with intensive research into the underlying pathogenesis and the development of biologics. While biologics for Th2-high asthma thrive, attempts targeting Th2-low asthma (e.g., brodalumab, a biologic targeting the IL-17 receptor) have failed due to pathogenic complexity and biomarker scarcity (Busse et al., 2013). Currently, the FDA has approved six biologics targeting Th2 inflammatory cascade effectors, including Omalizumab targeting IgE, Dupilumab targeting IL-4R, Mepolizumab, Benralizumab, Reslizumab targeting IL-5, and Tezepelumab targeting TSLP (Bourdin et al., 2024). Table 1 lists the current treatment measures for asthma.

**TABLE 1:** Current therapeutic approaches for the management of asthma.

Clinical therapies	Drugs	Targets	Mechanisms
Conventional therapies	Glucocorticoids: Beclomethasone Dipropionate, Hydrocortisone, Dexamethasone	GR	Interfering with arachidonic acid metabolism, inhibiting eosinophil chemotaxis and activation, suppressing the synthesis of pro-inflammatory cytokines, reducing microvascular leakage, and increasing the synthesis of *β*2-receptors on cell membranes
	Beta-2 Adrenergic Agonists: Clenbuterol, Salmeterol, Formoterol	*β*2R	Relaxing bronchial smooth muscle, reducing inflammatory mediator release, decreasing microvascular permeability, and enhancing ciliary movement of airway epithelium
	Cholinergic Receptor Antagonists: Ipratropium Bromide, Tiotropium Bromide, Glycopyrronium Bromide	M3R	Inhibiting the action of acetylcholine, reducing contraction of airway smooth muscle
	Leukotriene Receptor Antagonists: Montelukast, Zafirlukast	CysLT1	Dilating the airways, preventing and reducing mucosal inflammatory cell infiltration
	Xanthine Derivatives: Aminophylline, Dyphylline, Oxtriphylline	PDE, AR	Relaxing bronchial smooth muscle, stimulating the respiratory center and respiratory muscles, and exerting anti-inflammatory effects
Biologic Therapies	Omalizumab	IgE	Binding to IgE, reducing serum-free IgE levels, downregulating high-affinity IgE receptors (Fc*ε*RⅠ) on immune cells, thereby inhibiting IgE binding to effector cells
	Dupilumab	IL-4R*α*	Blocking the signaling of IL-4 and IL-13, reducing Th2-type inflammatory responses
	Mepolizumab	IL-5	Targeting and binding to human IL-5, preventing the binding of IL-5 to the α chain of its receptor complex on eosinophils, thereby inhibiting IL-5’s biological activity
	Reslizumab		
	Benralizumab	IL-5R*α*	By high-affinity binding to IL-5Rα on eosinophils, preventing their interaction with IL-5 and inducing eosinophil apoptosis through antibody-dependent cell-mediated cytotoxicity, thus reducing eosinophil levels
	Tezepelumab	TSLP	Blocking the interaction of TSLP with its receptor, inhibiting TSLP-driven Th2 cell differentiation and Th2 inflammatory cytokine release

Significant shortcomings exist in current asthma treatments. Prolonged use of corticosteroids can lead to various local side effects such as dysphonia, candidiasis, etc., and systemic side effects such as osteoporosis, cataracts, etc. There are short- and long-term risks associated with *β*_2_-agonist use, including drug tolerance and adverse cardiovascular effects. The latest immunotherapy, however, is affected by its limitations and high cost and is used as an adjunct to corticosteroids rather than an alternative treatment (Cevhertas et al., 2020). Natural medicines have garnered significant interest due to their high efficiency and low toxicity, and this paper will focus on the advantages of CHM in asthma treatment.

## TRPV1/TRPA1 channels

Transient receptor potential (TRP) channels are a class of non-selective cation channels involved in the regulation of sensory transduction in living organisms as well as cellular physiopathological processes. As polymodal channels, TRP channels act as “signal sensors” or “signal detectors” for the organism, sensing and responding to a variety of environmental and physiological stimuli, including temperature, electrical stimuli, and endogenous or exogenous chemical substances (Diver et al., 2022). Ca^2+^ is a crucial intracellular second messenger. Upon activation by stimuli, TRP channels mediate the direct influx of Ca^2+^, generating action potentials and subsequently regulating numerous biological processes, including cell contraction, proliferation, migration, secretion, and apoptosis (Thakore and Earley, 2019).

Since the first TRP channel was discovered in fruit flies in 1969 (Cosens and Manning, 1969), researchers have identified 28 members of the TRP channel superfamily in mammals, which are divided into two groups based on sequence homology, encompassing seven subfamilies. The first group includes TRPC, TRPV, TRPM, TRPA, and TRPN, while the second group consists of TRPP and TRPML, with each subfamily containing one or more subsets (Zhang et al., 2023). Systematic analyses using various techniques have shown that TRP channels are primarily tetrameric structures composed of six transmembrane domains (S1–S6), with a pore-forming region between S5 and S6 that enables ion passage. The differences among family members mainly reside in the structures at the intracellular N- and C-termini (Gaudet, 2008). TRP channels are widely distributed in organs such as the nervous system, heart, lungs, liver, kidneys, intestines, and skin, participating in various physiological activities and pathological developments and associating with a broad range of diseases, making them attractive therapeutic targets (Zhang et al., 2023). Moreover, numerous studies have revealed that TRP channels, particularly TRPV1 and TRPA1, are extensively present in the respiratory system, functioning as “gatekeepers of inflammatory diseases”. They are involved in airway sensory transduction and inflammation regulation, playing a significant role in the pathophysiology of asthma (Banner et al., 2011; Bautista et al., 2013; De Logu et al., 2016). In 1997, TRPV1 was first cloned from rat dorsal root ganglion (DRG) (Caterina et al., 1997). The TRPV1 channel was primarily expressed in unmyelinated C-type and myelinated A*δ*-type sensory nerve fibers within the dorsal root ganglia and trigeminal ganglia. They are also expressed throughout the respiratory system (including the upper airway, lower airway, and alveoli) and can be activated by various endogenous or exogenous stimuli, such as high temperatures (>43°C), low pH, vanilloid compounds, and pro-inflammatory cytokines (Watanabe et al., 2005; Romanovsky et al., 2009; Zhang et al., 2023). Additionally, TRPV1 expression has been detected in some immune cells (McGarvey et al., 2014; Xiao et al., 2022). TRPA1 is initially cloned from human lung fibroblasts (Jaquemar et al., 1999). Further research has shown that TRPA1 is widely distributed in neurons of the dorsal root ganglia, trigeminal ganglia, and vagal ganglia, often co-expressed with TRPV1 (Jaquemar et al., 1999). In the respiratory system, TRPA1 is also expressed in airway epithelial cells, fibroblasts, and smooth muscle cells. It is involved in detecting temperature [cold (<17°C)] and a variety of exogenous and endogenous chemical substances. These include exogenous stimuli such as cinnamaldehyde, acrolein, garlic, allyl isothiocyanate, and industrial pollutants, as well as endogenous stimuli such as reactive oxygen species (ROS), prostaglandins (PG), nerve growth factor (NGF), bradykinin (BK), lipid peroxides, etc. (Koivisto, 2012; Talavera et al., 2020).

An increasing number of studies have demonstrated the potential of natural metabolites targeting TRP channels in the treatment of airway diseases (Liu et al., 2021; Stinson et al., 2023). Therefore, it is likely that promising asthma therapies can be developed based on natural products or CHM.

## The role of TRPV1/TRPA1 channels in asthma

**FIGURE 2:**
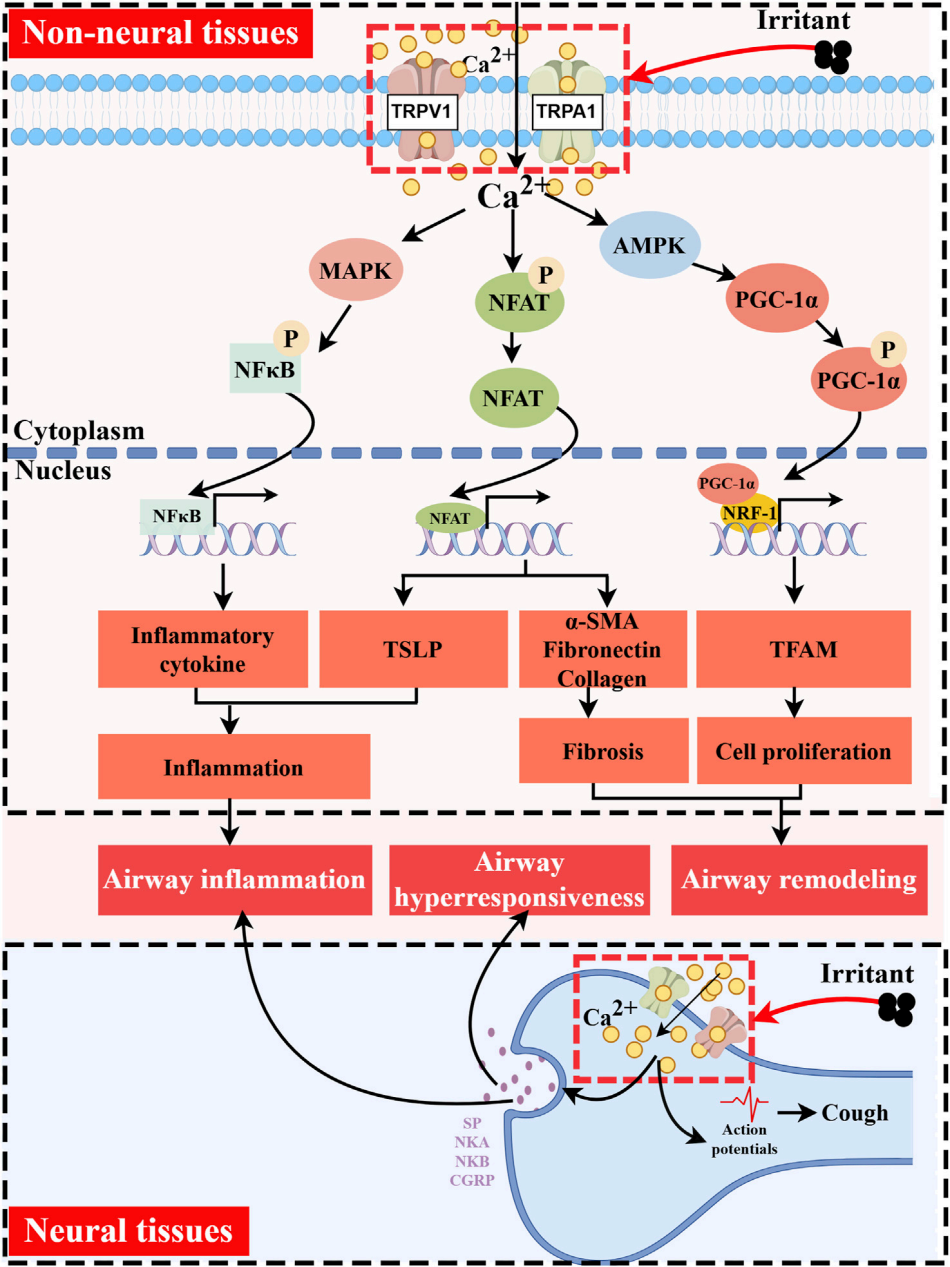
Functions of TRPV1/TRPA1 in Asthma. TRPV1/TRPA1 channels are activated by temperature, mechanical forces, and diverse endogenous and exogenous chemical stimuli. They mediate Ca^2+^ influx and participate in key pathological processes of asthma, including airway inflammation, airway hyperresponsiveness, and airway remodeling. In non-neuronal tissues, Ca^2+^ signaling promotes PGC-1α phosphorylation via AMPK, which in turn cooperates with NRF-1/TFAM to initiate mitochondrial biogenesis, ultimately leading to cell proliferation. Concurrently, elevated intracellular Ca^2+^ induces the dephosphorylation and nuclear translocation of NFAT, regulating gene expression associated with cellular fibrosis and immune responses. Additionally, increased intracellular Ca^2+^ can activate the MAPK pathway, facilitating nuclear translocation of NF-κB to drive the transcription of inflammatory genes. In neuronal tissues, Ca^2+^ influx triggers the release of neuropeptides such as SP, NKA, NKB, and CGRP. These neuropeptides directly induce airway smooth muscle contraction and mucus hypersecretion, while also contributing to the activation and recruitment of immune cells. Created with figdraw.com.

Alleviating or even curing this inflammation is key to asthma prevention and treatment. Studies have shown that polymorphisms in the TRPV1 and TRPA1 are closely associated with the pathophysiological processes of asthma (Cantero-Recasens et al., 2010; Gallo et al., 2017). These channels are involved in sensory transduction and the regulation of neurogenic and immunogenic inflammation during asthma development. TRPV1 and TRPA1 can directly or indirectly affect intracellular pathways, thereby activating or inhibiting inflammatory responses. This process involves crosstalk among various cell types, including neurons, immune cells, airway epithelial cells, and others. Here, we summarize the pathological roles of TRPV1/TRPA1 in asthma (Figure 2).

### TRPV1/TRPA1 expression and functional roles in neuronal and non-neuronal tissues

TRPV1 and TRPA1 are predominantly localized in sensory C fibers, where they are co-expressed in neurons, with TRPA1 often accompanying TRPV1. However, neurons expressing TRPV1 do not always fully co-express TRPA1. These channels demonstrate significant positive interaction when co-activated, playing crucial roles in regulating both normal physiological and pathological functions of the respiratory tract (Nassenstein et al., 2008; Lin et al., 2015b). During the course of asthma, TRPV1/TRPA1-mediated calcium influx induces the release of neuropeptides, including SP, NKA, NKB, and CGRP. These neuropeptides directly contribute to airway hyperresponsiveness and amplify inflammatory responses (Zhang et al., 2023). Consequently, antagonism of TRPV1/TRPA1 channels exerts beneficial effects on asthma symptoms. For instance, the use of TRPV1 or TRPA1 antagonists has been found to significantly inhibit histamine-induced airway hyperreactivity in ovalbumin (OVA)-sensitized guinea pigs without affecting pulmonary inflammation in the short term (Delescluse et al., 2012; van den Berg et al., 2021).

TRPV1 and TRPA1 are also present in non-neuronal tissues of the lung. TRPV1 is distributed in the airway epithelium, and it is involved in bronchoconstriction, abnormal proliferation of airway smooth muscle, and airway remodeling. A study analyzing bronchial biopsy and brush samples from healthy volunteers, patients with mild or moderate asthma, and patients with severe asthma revealed the presence of functional TRPV1 in the human airway epithelium, with significant overexpression observed in the airways of patients with severe asthma (McGarvey et al., 2014). Furthermore, activation of TRPV1 initiates mitochondrial biogenesis via the PGC-1α/NRF-1/TFAM pathway, promoting the proliferation of airway smooth muscle cells (Li et al., 2023). Conversely, inhibition of this channel effectively attenuates cell proliferation and alleviates airway remodeling, characterized by goblet cell hyperplasia and collagen deposition (Zhao et al., 2013; Choi et al., 2018). It was found that lysophosphatidic acid (LPA) stimulation of the carotid body can cause reflex stimulation of vagal efferent activity, leading to acute bronchoconstriction in OVA-sensitized rat model (Jendzjowsky et al., 2018). Their subsequent research indicated that LPA and asthma-associated Th2 cytokines mediate this phenomenon by inducing PKC*ε*-dependent phosphorylation at residues T704 and S502 of the TRPV1 channel in the carotid body. Notably, blocking PKC*ε* or TRPV1 was shown to inhibit airway hyperreactivity in the OVA-sensitized rat model without affecting the carotid body’s response to hypoxic reflexes. This finding suggested a potential new target for treating asthma-related bronchial hyperreactivity (Jendzjowsky et al., 2021a; Jendzjowsky et al., 2021b). Furthermore, the airway epithelium, including fibroblasts and smooth muscle cells, functionally expresses TRPA1, which mediates airway narrowing by mobilizing intracellular and extracellular Ca^2+^, thereby inducing airway hyperresponsiveness (Vaghasiya et al., 2023). TRPA1 can also promote tissue fibrosis by regulating the production of fibronectin, collagen, and α-smooth muscle actin (α-SMA) via NFAT signaling, a process that may contribute to airway remodeling (Yap et al., 2021). It is also noteworthy that both TRPV1 and TRPA1 can activate the MAPK pathway, facilitating NF-κB nuclear translocation to drive the transcription of inflammatory genes, ultimately leading to airway inflammation (Liu et al., 2020).

TRPV1/TRPA1 mediates the crosstalk between the nervous and immune systems, influencing both acute and chronic symptoms of asthma. TRPV1 is functionally expressed in CD4^+^ T cells, where it plays a role in T cell receptor signaling, activation, and the release of inflammatory cytokines (Bertin et al., 2014). TRPA1 has also been found to be highly expressed in CD4^+^ T cells within the lung tissue of C57BL/6 mouse asthma models, with expression levels increasing with asthma severity (Li et al., 2020). Moreover, CGRP released from TRPV1^+^ neurons inhibited neutrophil recruitment and surveillance functions, altered the number of pulmonary *γδ* T cells, and mediated immunosuppression (Baral et al., 2018).

### Exogenous and endogenous modulation of TRPV1/TRPA1 in asthma pathogenesis

A growing body of research suggests that environmental factors are associated with the onset and exacerbation of asthma. Firstly, environmental pollutants are closely linked to the high prevalence of asthma. Numerous studies have demonstrated that exposure to pollutants such as PM2.5, ozone (O_3_), nitrogen dioxide (NO_2_), carbon black nanoparticles (CB NPs), trimellitic anhydride (TMA), toluene-2,4-diisocyanate (TDI), hypochlorite, and cigarette smoke can induce increasing expression of TRPV1 or TRPA1 in the lungs of mouse asthma models. This exposure can also enhance the production of neuropeptides like SP and CGRP, leading to elevated airway inflammation and oxidative stress. These changes result in increased airway hyperresponsiveness, mucus secretion, and subepithelial fibrosis, thereby exacerbating various asthma symptoms (Hox et al., 2013; Devos et al., 2016; Harford et al., 2021; Sun et al., 2021; Deng et al., 2022; Lian et al., 2022; Lu et al., 2023). Secondly, environmental temperature and humidity are potential asthma triggers. Hyperventilation in warm, humid air increases the excitability of C fibers, which further enhances the rats’ response to capsaicin (Lin et al., 2015a). A study found that exposure to high (40°C) or low (10°C) temperatures elevated levels of inflammatory cells and cytokines in the bronchoalveolar lavage fluid of Balb/c mouse asthma models. This exposure significantly upregulated TRPV1 or TRPA1 in lung tissue, exacerbating asthma-like pathological features, which could be alleviated by using TRPV1 antagonists such as capsazepine or TRPA1 antagonists like HC030031 (Du et al., 2019; Lu et al., 2023; Deng et al., 2024). Notably, compared to the pathogenic effects of a single pollutant, combined exposure to multiple pollutants creates a synergistic effect that significantly increases the incidence and severity of asthma in a dose-dependent manner. Finally, compared to the effects produced by the direct activation of TRP channels by exogenous stimuli, endogenous factors may play a more crucial role in the chronic progression of asthma in patients. On the one hand, pulmonary inflammation leads to the release of endogenous TRP agonists—including arachidonic acid metabolites, bradykinin, and endogenous aldehydes—which activate TRP channels and exacerbate disease symptoms. On the other hand, during asthma progression, inflammation regulates the expression levels of TRP channels and lowers their activation threshold, thereby inducing disease deterioration (Hwang et al., 2000; Koivisto, 2012). For example, the impact of inflammatory factors on the expression and function of TRPA1 in human lung epithelial cells was that IL-4 and IL-13 inhibited TRPA1 expression via the JAK/STAT6 pathway, while IFN-*γ* upregulated TRPA1 expression (Luostarinen et al., 2023). Besides, endogenous inflammatory mediators such as bradykinin and trypsin can act on specific G protein-coupled receptors, activating downstream phospholipase C (PLC), which degraded phosphatidylinositol 4,5-bisphosphate (PIP2) in the cell membrane. This degradation removed inhibition on TRPV1/TRPA1, rendering the channels sensitized (Chuang et al., 2001; Dai et al., 2007).

### Therapeutic targeting paradoxes and dual roles of TRPV1/TRPA1 activation

Recently, the role of TRPV1/TRPA1 activation in exacerbating asthma symptoms and its specific mechanisms have become increasingly clear, sparking interest among asthma drug development researchers in targeting TRPV1/TRPA1 antagonists. However, some studies have presented findings that contradict these insights. Capsaicin, a common TRPV1 agonist, was found to induce the maturation of DCs through TRPV1 activation (Basu and Srivastava, 2005). In contrast, it was observed that capsaicin inhibited the differentiation, maturation, and pro-inflammatory cytokine release of DCs in a dose-dependent manner (Tóth et al., 2009). Moreover, knocking out TRPV1 in house dust mite (HDM) or OVA-sensitized asthma mouse models led to an enhanced Th2 response (Mori et al., 2011). Additionally, TRPA1 activation may also have beneficial effects. Allyl isothiocyanate, a natural TRPA1 agonist, was shown to protect airway barriers, reduce airway inflammation, suppress airway hyperreactivity, and alleviate symptoms related to allergic asthma in rat models (Lai et al., 2023). These experimental observations suggest that the activation of TRPV1 and TRPA1 might attenuate Th2 responses and inhibit the progression of airway inflammation, indicating that TRPV1 and TRPA1 may have dual roles in the pathogenesis of asthma.

## Chinese herbal medicines for asthma treatment

**FIGURE 3:**
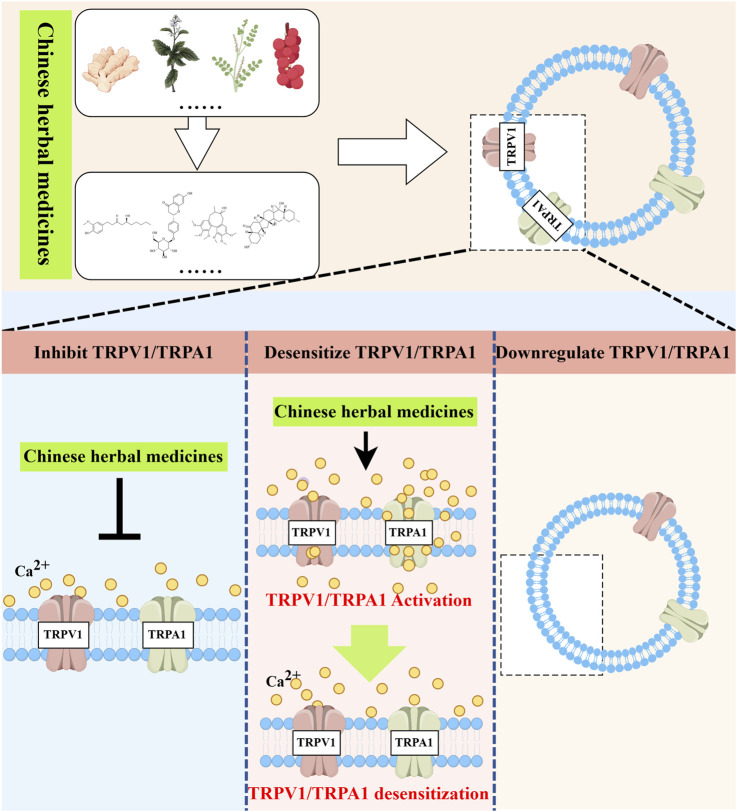
Natural medicines treat asthma with TRPV1/TRPA1. Natural herbal medicines suppress airway inflammation and airway hyperresponsiveness and ameliorate asthma symptoms by either directly inhibiting TRPV1/TRPA1, inducing receptor desensitization, or indirectly downregulating the expression of TRPV1/TRPA1. Created with figdraw.com.

Over the years, the prevalence has remained high despite the diversification of strategies for treating asthma. Consequently, the development and application of new asthma drugs continue to be a cutting-edge research focus. Botanical drug and their monomeric metabolites, abundant in nature, serve as excellent sources of medication, offering rich resources with minimal side effects. These metabolites may alleviate asthma symptoms by modulating TRPV1/TRPA1 (Figure 3).

### Chinese herbal formulas

CHM has a long history and unique advantages in treating asthma. Under the guidance of traditional Chinese medicine theoretical systems, various combinations of botanical drugs are used to form complex herbal formulations. San’ao Decoction (SAD), originating from the “Taiping Benevolent Prescriptions,” is composed of Herba Ephedrae, Semen Armeniacae Amarae, and Radix Glycyrrhizae. It has the functions of dispersing lung cold, stopping coughing, and relieving asthma, making it a classic formula for treating asthma throughout history. It was found that SAD inhibits IL-4-induced apoptosis of normal human bronchial epithelial cells, thereby offering protective effects on these cells (Li et al., 2010). Their subsequent studies suggested that SAD might participate in immunoregulation by increasing the proportion of CD4^+^CD25^+^Foxp3^+^ Treg cells in the spleen of mice, thereby alleviating airway hyperresponsiveness and inflammatory cell infiltration in OVA-sensitized asthma mouse models (Li et al., 2014). Further investigation showed that SAD can inhibit TRPA1/TRPV1 channels, reduce airway inflammatory factors (IL-13, PGD2, and NGF) levels, improve lung function in OVA + PM2.5 exacerbated asthma mouse models, and alleviate lung injury (Wang et al., 2019). Additionally, the combination of SAD with scorpio and bombyx batryticatus significantly alleviated neurogenic inflammation and cough frequency in CVA mouse models (Wang et al., 2021). Computer technology is of significant importance for research on the mechanisms of CHM, which is characterized by multiple metabolites and targets. Twenty-eight chemical metabolites were identified in SAD using UPLC-Q-TOF/MS, and three main metabolites (ephedrine, amygdalin, and Enoxolone) were selected for computer-assisted analysis and experimental validation. These metabolites were demonstrated to exert therapeutic effects on asthma by binding to the D509 and D647 sites of the TRPV1 channel and regulating Ca^2+^ influx (Jiang et al., 2024). Of course, both virtual screening and *in vitro* model validation have inherent limitations, necessitating additional *in vivo* studies for verification. Nevertheless, this integrated approach provides valuable insights into CHM’s complex mechanisms while addressing its multi-metabolite nature.

Ma Xin Shi Gan Decoction (MXSGD) is documented in the “Treatise on Cold Damage Diseases” and is composed of four medicines: Herba Ephedrae, Semen Armeniacae Amarum, Gypsum Fibrosum, and Radix Glycyrrhizae Preparata. MXSGD reduced the levels of IL-4, IL-13, PGE2, and SP in the bronchoalveolar lavage fluid of RSV-exacerbated asthma mouse models (Li et al., 2022b). It downregulated the expression of TRPV1 in lung tissue and decreased the capsaicin-induced increase in Ca^2+^ concentration in 16HBE cells, improving airway inflammation and hyperresponsiveness (Li et al., 2022b).

She Gan Ma Huang Decoction (SGMHD), originating from the “Synopsis of Prescriptions of the Golden Chamber”, consists of Rhizoma Belamcandae, Herba Ephedrae, Rhizoma Zingiberis Recens, Herba Asari, Radix Asteris, Flos Farfarae, Fructus Jujubae, Rhizoma Pinelliae, Fructus Schisandrae. SGMHD was shown to alleviate airway inflammation and mitigate airway remodeling in cold-induced asthma rats by regulating the TRPV1/NRF-1/mtTFA signaling pathway (Li et al., 2023). The acrid and bitter botanical drugs in SGMHD might be involved in the regulation of the TRPV1 channel (Yuan et al., 2022).

Hou Pu Ma Huang Decoction (HPMHD), originating from the “Synopsis of Prescriptions of the Golden Chamber,” consists of Magnolia officinalis, Herba Ephedrae, Gypsum Fibrosum, Semen Armeniacae Amarae, Herba Asari, Rhizoma Zingiberis, Fructus Schisandrae, Rhizoma Pinelliae, and Fructus Tritici levis. HPMHD reduced lung inflammation and airway hyperresponsiveness in cigarette smoke-induced exacerbated asthma mouse models by downregulating TRPA1 and upregulating the expression of tight junction proteins in airway epithelium. Subsequently, it was found that TRPV1 may also be involved in HPMHD’s anti-asthma effects (Sun et al., 2022; Zhou et al., 2023). Additionally, other herbal formulations are listed in Table 2.

**TABLE 2:** List of Chinese herbal formulas with potential anti-asthmatic effects via TRPV1/TRPA1 regulation.

Herbal formulas	Composition	Model	Positive control	Dosage	Biological activity	References
San’ao Decoction	Herba Ephedrae, Semen Armeniacae Amarae, and Radix Glycyrrhizae	Female BALB/c mice	Dexamethasone (0.75 mg/kg)	0.9, 1.8 g/kg	Inhibition of TRPA1/TRPV1 channels, protection of bronchial epithelial cells, involvement in immune regulation, alleviation of airway inflammation, and reduction of cough frequency	Wang et al. (2019)
Ma Xing Shi Gan Decoction	Herba Ephedrae, Semen Armeniacae Amarum, Gypsum Fibrosum, and Radix Glycyrrhizae Preparata	Female C57BL/6 mice	Dexamethasone (0.75 mg/kg)	3.3, 6.6, 13.2 g/kg	Reduction of TRPV1 expression, improvement of airway inflammation, and reduction of airway hyperresponsiveness	Li et al. (2022b)
		16HBE				
She Gan Ma Huang Decoction	Rhizoma Belamcandae, Herba Ephedrae, Rhizoma Zingiberis Recens, Herba Asari, Radix Asteris, Flos Farfarae, Fructus Jujubae, Rhizoma Pinelliae, Fructus Schisandrae	Male SD rats	Dexamethasone (0.4 mg/kg)	3.2, 6.4, 12.8 g/kg	Reduction of TRPV1 expression, improvement of airway inflammation, and alleviation of airway remodeling	Li et al. (2023)
Hou Pu Ma Huang Decoction	Mangnolia officinalis, Herba Ephedrae, Gypsum Fibrosum, Semen Armeniacae Amarae, Herba Asari, Rhizoma Zingiberis, Fructus Schisandrae, Rhizoma Pinelliae, and Fructus Tritici levis	Female C57BL/6 mice	Dexamethasone (0.1 mg/kg)	6.0, 12.0 g/kg	Downregulation of TRPA1 and upregulation of airway epithelial tight junction protein expression, alleviation of pulmonary inflammation, and reduction of airway hyperresponsiveness	Sun et al. (2022)
Qingfei Oral Liquid	Herba Ephedrae, Semen Armeniacae Amarae, Gypsum Fibrosum, Radix Scutellariae, Cortex Mori, Rhizoma Acori Graminei, Radix Paeoniae Rubra, Rhizoma Polygoni Cuspidati, and Radix Salviae Miltiorrhizae	Male BALB/c mice	Dexamethasone (0.2 mg/kg)	6.365 g/kg	Reduction of TRPV1 expression, inhibition of mucus secretion, and alleviation of airway inflammatory infiltration	Jing et al. (2020)
Qufeng Zhijing Capsule	Bombyx Batryticatus, Periostracum Cicadae, Pberetima	Male Wistar rats	Budesonide (1.0 mg/kg)	0.2, 0.5, 1.6 g/kg	Downregulation of TRPV1 expression in airway smooth muscle, suppression of airway inflammatory response	Wu et al. (2024)
Majing Zhike Granule	Herba Ephedrae, Herba Schizonepetae, Periostracum Cicadae, Fructus Trichosanthis, Flos Inulae, Flos Farfarae, Radix Glehniae, Radix Glycyrrhizae	Male Hartley guinea pigs	Dexamethasone (2.0 mg/kg)	0.93 g/kg	Downregulation of TRPV1 expression in the lungs, reduction of airway sensitivity, and amelioration of airway inflammation	Chen and Cai (2022)
Jiuwei Maxing Granule	Herba Ephedrae, Semen Armeniacae Amarae, Radix Glycyrrhizae Preparata, Rhizoma Cynanchi Stauntonii, Radix Asteris, Flos Farfarae, Radix Stemonae, Radix Platycodi, Rhizoma Smilacis Glabrae	Male Hartley guinea pigs	-	6.4 g/kg	Downregulation of TRPV1 expression in the lungs, inhibition of neurogenic inflammation	Wang et al. (2023)
Baijiezi Tufa	Semen brassicae, Rhizoma Corydalis, Euphorbia Kansui, Herba Asari, Moschus	Male Hartley guinea pigs	-	0.05 g (topical application)	Downregulation of TRPV1 in the lungs, reduction of airway hyperresponsiveness	Li et al. (2017)

### Botanical drugs modulating TRP channels

#### TRPV1

Ginger (*Zingiber officinale* Rosc.) is a perennial botanical drug from the Zingiberaceae family, whose underground rhizomes are noted for their unique spicy and aromatic flavor. It is widely used in culinary and medicinal fields. Modern pharmacological studies show that active metabolites of ginger can inhibit the migration and proliferation of human bronchial smooth muscle cells induced by phthalates, thereby alleviating airway remodeling (Kuo et al., 2011). Besides, Ginger’s active metabolites may treat asthma through acute airway smooth muscle relaxation and chronic inflammation inhibition (Townsend et al., 2013). A study found that the main metabolites of ginger (6-shogaol, 6-gingerol, 8-gingerol) can rapidly relax pre-constricted isolated human airway smooth muscle. In subsequent studies, it was demonstrated that long-term administration of whole ginger extract or ginger’s bioactive metabolite (6-shogaol) can reduce lung inflammation in dust mite antigen-sensitized mouse asthma models by increasing cAMP concentrations in CD4^+^ cells and inhibiting NF-*κ*B signaling (Townsend et al., 2013; Yocum et al., 2020). Ginger contains various metabolites capable of binding to TRPV1. It was found that three characteristic metabolites (6-shogaol, 6-gingerol, zingerone) can activate TRPV1 (activity: 6-shogaol > 6-gingerol > zingerone), forming hydrogen bonds at residues T551 and E571, with binding sites consistent with capsaicin (Yin et al., 2019). TRPV1 activation typically triggers inflammation and pain, seemingly contradicting ginger’s anti-asthmatic mechanisms. However, a recent intriguing study revealed that 8-gingerol, an active metabolite in ginger, induces TRPV1 endocytosis and degradation after activation, thereby reducing sensitivity to acute and inflammatory pain. 8-gingerol was also compared with the conventional TRPV1 agonist capsaicin, showing fewer side effects and higher safety (Cheng et al., 2024). Consequently, the active metabolites of ginger may exert anti-asthmatic effects by mediating TRPV1 desensitization, revealing potential mechanisms for treating asthma with natural TRPV1 agonists.

The use of loquat leaves, *Eriobotrya japonica* (Thunb.) Lindl., to treat airway inflammation, coughs, and other respiratory diseases has a long history in Asia. It was reported that the water extract of loquat leaves significantly reduced the infiltration of inflammatory cells and mucus production in an OVA-sensitized mouse asthma model, as well as decreased levels of NO, EPO, IL-4, IL-13 in bronchoalveolar lavage fluid, and serum IgE levels (Kim et al., 2020b). Subsequently, *in vitro* experiments revealed that it also suppressed the proliferation of human tracheal smooth muscle cells and the expression of inflammatory pathways such as NF-*κ*B (Kim et al., 2020b). The main active metabolites of loquat leaves are likely to be total flavonoids. Total flavonoids from loquat leaves reduced the secretion of inflammatory cytokines in a cigarette smoke-induced mouse COPD model, downregulated the expression of TRPV1, cytochrome P450 2E1 (CYP2E1), and *p*-JNK in tissues, and upregulated the expression of SOD-2, exhibiting significant anti-inflammatory and antioxidant activities (Jian et al., 2020). The therapeutic effects of loquat leaves may be partially related to the inhibition of TRPV1 channel activation. A study comparing the regulatory effects of various herbal medicines extracts on TRP channels found that an ethanol extract of loquat leaves exhibited effective desensitization of TRPV1. The metabolite analysis identified ursolic acid as a significant inhibitor of TRPV1 activation (Zhang et al., 2011).

Fritillaria is a well-known cough suppressant in China. It was demonstrated that total alkaloids from *Fritillaria unibracteata* var. wabuensis bulbus could alleviate airway mucus secretion, collagen deposition, and inflammatory cell infiltration in an OVA-sensitized asthma mouse model by inhibiting the TRPV1/Ca^2+^/NFAT pathway (Peng et al., 2023). Furthermore, two metabolites, sipeimine and edpetiline were identified, which directly block TRPV1-mediated Ca^2+^ influx (Peng et al., 2023). The other two metabolites, Peiminine and Peimine, which were also identified from *Fritillaria cirrhosa* D. Don can significantly enhance the inhibitory effect of ursolic acid from loquat leaves on TRPV1, suggesting a rationale for the combined use of certain natural medicines (Zhang et al., 2011).

Sinomenine is an alkaloid extracted from the roots of *Sinomenium acutum* (Thunb.)Rehd. et Wils., known for its anti-inflammatory, analgesic, and antiviral activities. Recent studies have shown that sinomenine significantly reduces airway inflammation and remodeling in a mouse model of asthma (Bao et al., 2016). Sinomenine downregulated the expression of TGF-*β*1 and Smad3, reduced subepithelial collagen deposition, and inhibited epithelial-mesenchymal transition both *in vivo* and *in vitro* (He et al., 2021). In the respiratory system, sinomenine can inhibit the process whereby capsaicin stimulation increases SOX5 expression, which in turn mediates the transcriptional upregulation of TRPV1 (Ma et al., 2021).

#### TRPA1

Stemona root, which includes the species *Stemona sessilifolia* (Miq.) Miq., *Stemona japonica* (Bl.) Miq., and *Stemona tuberosa* Lour., is known for its ability to nourish lung qi, relieve cough, and eliminate parasites. It is a commonly used botanical drug in China for treating respiratory diseases. The water extract of *S. sessilifolia* dose-dependently (1–50 mg/mL) alleviated bronchial smooth muscle contraction induced by carbamylcholine, histamine, and KCl in guinea pigs (Liao et al., 1997). Furthermore, it was shown that the water extract of S. tuberosa reduced inflammatory responses in LPS-stimulated RAW 264.7 macrophages and in a cigarette smoke-induced mouse model of lung inflammation (Lim et al., 2015). Additionally, the main active metabolite of *S. tuberosa*, neotuberostemonine, alleviated the cough response in a mycoplasma pneumonia mouse model by downregulating the expression of TRPA1 and improving lung tissue structure (Liang et al., 2022). This finding suggested that the active metabolites in Stemona could also mitigate symptoms of cough-variant asthma by inhibiting TRPA1 channel expression.

Allyl isothiocyanate (AITC) is a TRPA1 agonist primarily found in plants of the Brassicaceae family, such as broccoli, cabbage, and mustard, as well as in botanical drugs like Radish Seed (*Raphanus sativus* L.) and Nux Vomica Seed (*Strychnos nux-vomica* L.). It was discovered that AITC-induced airway bronchodilation in a dose-dependent manner through organ bath experiments on guinea pigs, mice, and human airways (Marsh et al., 2020). AITC downregulated TRPA1 protein levels, increased the expression of tight junction-related proteins, reduced airway inflammation, and alleviated symptoms in an HDM-induced allergic asthma model (Lai et al., 2023). Additionally, other studies have reported that AITC can inhibit airway remodeling by downregulating the expression of *α*-smooth muscle actin in TGF-*β*1-treated lung fibroblasts via the ERK1/2 MAPK and NRF2/HO-1 pathways, a process that was independent of TRPA1 regulation (Yap et al., 2021).

Menthol is a natural TRPM8 agonist derived from *Mentha haplocalyx* Briq. (Lamiaceae family), and it can also modulate TRPA1. Nebulized inhalation of peppermint essential oil, whose main metabolites are menthol and menthone, can inhibit airway epithelial hyperplasia, collagen deposition, and goblet cell activation in PM10-exposed asthmatic mice (Kim et al., 2020a). Nebulized inhalation of menthol alone can also significantly reduce airway inflammation and decrease airway hyperresponsiveness in asthma (Wang et al., 2018). Some studies indicate that menthol can activate TRPA1, inhibiting the proliferation of ASMCs and alleviating airway remodeling in asthma patients (Zhang et al., 2016). However, menthol can also induce asthma responses to some degree, possibly due to a biphasic effect of TRPA1 by menthol. Specifically, low concentrations of menthol mediate channel activation, whereas higher concentrations may induce reversible channel blockade (Karashima et al., 2007). Further research is needed to determine the appropriate dosing and whether menthol can be effectively used in asthma treatment.

#### TRPV1/TRPA1

In CHM formulations, licorice (Roots of *Glycyrrhiza uralensis* Fisch.) is a commonly used adjunctive botanical drug known for enhancing the efficacy of combined medications and improving clinical outcomes. Additionally, when used alone, licorice has expectorant, antiasthmatic, and lung-moistening properties, making it a common treatment for asthma and other respiratory diseases. The primary flavonoid active metabolite in licorice, liquiritigenin, downregulated the high expression of TRPV1 and TRPA1 proteins in lung tissue in an LPS-induced acute lung injury model, thereby reducing airway inflammation and cough response. Liquiritigenin can also inhibit the whole-cell currents of TRPV1 and TRPA1 induced by capsaicin and allyl isothiocyanate *in vitro* (Liu et al., 2020).

The fruit of *Schisandra chinensis* (Turcz.) Baill. is a medicinal resource widely used to treat respiratory diseases such as lung inflammation and cough. However, its active metabolites have not yet been fully understood. Comparing the anti-inflammatory effects of different fractions of Schisandra ethanol extract, including petroleum ether, ethyl acetate, and highly polar fractions, researchers found that the petroleum ether fraction exhibited significant anti-inflammatory effects in an OVA-induced asthma mouse model (Li et al., 2022a). Lignans are likely the effective metabolites in the treatment of asthma with Schisandra, possibly involving TRP channels. A study compared the effects of Schisandra lignans and polysaccharides on the tension of isolated rat tracheal smooth muscle and found that lignan metabolites showed a dose-dependent inhibitory effect on the tension of tracheal rings pre-contracted with ACh or KCl (Dong et al., 2023). Additionally, pretreatment with 1 g/kg of Schisandra ethanol extract significantly alleviated airway inflammation and hyperresponsiveness induced by cigarette smoke in guinea pigs and reduced TRPV1 and TRPA1 expression. Subsequently, four lignan metabolites (schisandrin, schisandrin A, deoxyschisandrin, and *γ*-schisandrin) were identified in the ethanol extract, which significantly inhibited the expression of TRPV1, TRPA1, NOS3, and the release of NO in A549 cells exposed to cigarette smoke extract (Zhong et al., 2015). Details of the anti-asthmatic activity of botanical drugs mediated through TRPV1/TRPA1 regulation are provided in Table 3, while those of CHM metabolites are summarized in Table 4.

**TABLE 3:** List of botanical drugs with potential anti-asthmatic effects mediated by TRPV1/TRPA1 regulation.

Botanical drugs	Extraction solution	Model	Positive control	Dosage	Biological activity	References
*Zingiber officinale* Ros.c	Supercritical CO2	C57BL/6 mice	—	40 mg/kg	Promotion of Treg polarization in CD4 cells, and suppression of inflammatory response	Yocum et al. (2020)
*Eriobotrya japonica* (Thunb.) Lindl.	Water	Female BALB/c mice	Montelukast (30 mg/kg)	50, 100, 200 mg/kg	Alleviation of airway inflammation cell infiltration and mucus production, and reduction of airway smooth muscle cell proliferation	Kim et al. (2020b)
		HTSMC; RAW 264.7				
*Fritillaria unibracteata* var. wabuensis bulbus	Chloroform: methanol mixture (4: 1, v: v)	Female C57BL/6 J mice	Dexamethasone (2.0 mg/kg)	100, 300 mg/kg	Inhibition of airway mucus secretion, collagen deposition, and inflammatory cell infiltration; reduction in TSLP production	Peng et al. (2023)
*Stemona tuberosa* Lour.	Water	C57BL/6 mice	Dexamethasone (1.0 mg/kg)	100 mg/kg	Inhibition of airway inflammatory	Lim et al. (2015)
		RAW 264.7				
*Mentha haplocalyx* Briq.	Hydrodistillation	Female BALB/c mice	Dexamethasone (2.0 mg/kg)	0.1 v/v %	Inhibition of airway epithelial hyperplasia, collagen deposition, and goblet cell activation	Kim et al. (2020a)
		A549				
*Schisandra chinensis* (Turcz.) Baill	Ethanol	Male C57BL/6J mice	Dexamethasone (2.0 mg/kg)	200, 400 mg/kg	Amelioration of airway inflammation	Li et al. (2022a)
		BEAS-2B				

**TABLE 4:** Lists of CHM Identified metabolites targeting TRPV1/TRPA1 for potential anti-asthmatic effects.

Targets	Metabolites	Source	Structure	Animals/Cells	Dosage	Positive control	Biological activity	References
TRPV1	6-shogaol	*Zingiber officinale* Rosc. [Zingiberaceae, Zingiberis rhizoma recens]	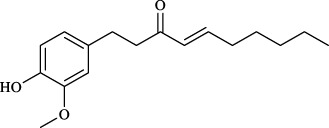	C57BL/6 mice	4.9 mg/kg	—	Promotion of Treg polarization in CD4 cells, and suppression of inflammatory response	Yocum et al. (2020)
	Ursolic acid	*Eriobotrya japonica* (Thunb.) Lindl. [Rosaceae, Eriobotryae folium]	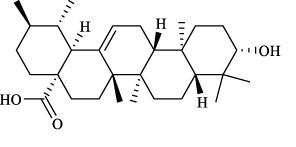	HEK 293T	100 μM	—	Cough Suppression	Zhang et al. (2011)
	Sipeimine	*Fritillaria unibracteata* var. *wabuensis* bulbus [Liliaceae, Fritillariae Cirrhosae Bulbus]	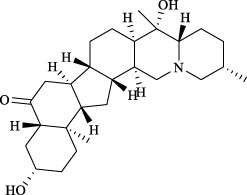	HEK 293T	3 nM	—	Inhibition of airway mucus secretion, collagen deposition, and inflammatory cell infiltration; reduction in TSLP production	Peng et al. (2023)
	Edpetiline		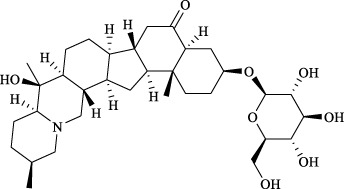		3 nM	—		
	Sinomenine	*Sinomenium acutum* (Thunb.) Rehd. et Wils. [Menispermaceae, Sinomenii Caulis]	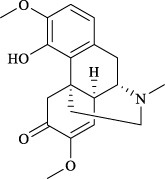	Female BALB/c mice	25, 50, 75 mg/kg	Dexamethasone (2.0 mg/kg)	Inhibition of airway inflammation, reduction of subepithelial collagen deposition, and suppression of epithelial-mesenchymal transition	Bao et al. (2016)
	Paeoniflorin	*Paeonia lactiflora* Pall. [Paeoniaceae, Paeoniae Radix Alba]	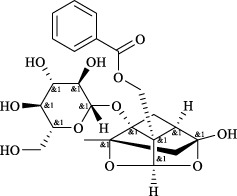	Female C57BL/6 mice	10, 25, 50 mg/kg	Dexamethasone (2.0 mg/kg)	Inhibition of oxidative stress and autophagy	Han et al. (2022)
	Imperatorin	Saposhnikovia divaricata (Turcz.) Schischk. [Apiaceae, Saposhnikoviae Radix]	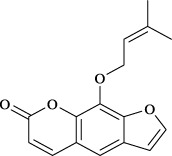	Female BALB/c and C57BL/6 mice	0.1, 1.0, 5.0 mg/kg	—	Suppression of Th2 cell activation	Lin et al. (2016)
	Resveratrol	Vitis amurensis Rupr. [Vitaceae]	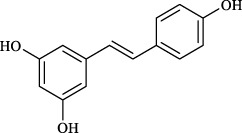	Female BALB/c mice	50 mg/kg	Dexamethasone (1.0 mg/kg)	Suppression of Syk protein expression and degranulation in mast cells, attenuating fibrotic responses	Chen et al. (2015)
				RBL-2H3				
	Osthole	*Cnidium monnieri*(L.)Cuss. [Apiaceae, Cnidii Fructus]	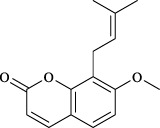	Female BALB/c mice	5.0, 25 50 mg/kg	—	Inhibition of activated CD4 T cell proliferation and Th1/Th2 cytokine production	Chiang et al. (2017)
	Quercetin	Multi-herb origin	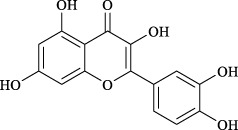	Male Wistar rats	50 mg/kg	Dexamethasone (2.5 mg/kg)	Inhibition of oxidative stress and inflammatory responses	Rajizadeh et al. (2023)
	Curcumin	*Curcuma Longa* L. [Zingiberaceae, Curcumae Longae Rhizoma]	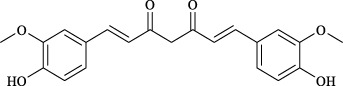	Female BALB/c mice	20 mg/kg	Dexamethasone (1.0 mg/kg)	Impact on the morphology and function of DCs	Yang et al. (2017)
	Baicalin	*Scutellaria baicalensis* Georgi [Lamiaceae, Scutellariae Radix]	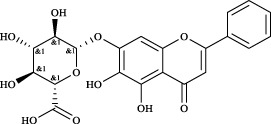	Female BALB/c mice	20 mg/kg	Dexamethasone (1.0 mg/kg)	Suppression of TSLP expression and type 2 immune responses	Zeng et al. (2024)
TRPA1	Neotuberostemonine	*Stemona tuberosa* Lour. [Stemonaceae, stemonae Radix]	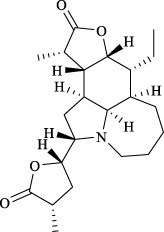	Male C57BL/6 mice	1.2 mg/kg	—	Inhibition of bronchial smooth muscle contraction, alleviation of pulmonary inflammation, and cough response	Liang et al. (2022)
	Allyl isothiocyanate	Multi-herb origin	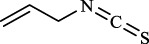	Female SD rats	10, 50, 100 mg/kg	Dexamethasone (1.0 mg/kg)	Alleviation of airway inflammation and pulmonary fibrosis; bronchodilation	Lai et al. (2023)
				BEAS-2B				
	Menthol	*Mentha haplocalyx* Briq. [Lamiaceae, Menthae Haplocalycis Herba]	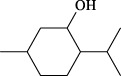	Female SD rats	300 *μ*M	—	Inhibition of ASMC proliferative phenotype	Zhang et al. (2016)
				ASMCs				
	Thymol	*Thymus vulgaris* [Lamiaceae]	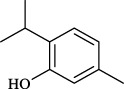	Female BALB/c mice	4, 8, 16 mg/kg	Dexamethasone (2.0 mg/kg)	Inhibition of NF-*κ*B activation	Zhou et al. (2014)
	Carvacrol	Multi-herb origin	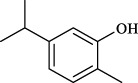	Male Wistar rats	15 mg/kg	Dexamethasone (1.0 mg/kg)	Inhibition of oxidative stress and inflammatory responses	Ezz-Eldin et al. (2020)
	Geraniol	Multi-herb origin	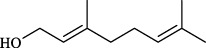	Female BALB/c mice	100, 200 mg/kg	—	Modulation of TH1/TH2 balance and inhibition of oxidative stress	Xue et al. (2016)
TRPV1/TRPA1	Liquiritin	*Glycyrrhiza uralensis* Fisch. [Fabaceae, Glycyrrhizae Radix et Rhizoma]	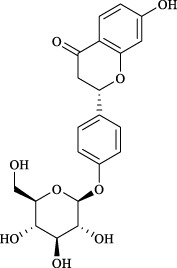	Male C57BL/6J mice	25, 50 or 100 mg/kg	—	Alleviation of airway inflammation and cough response	Liu et al. (2020)
				HEK 293T				
	Schizandrin	*Schisandra chinensis* (Turcz.) Baill. [Schisandraceae, Schisandrae Chinensis Fructus]	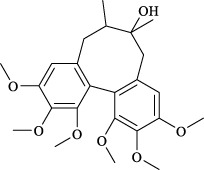	A549	100 *μ*M	—	Inhibition of TRPV1/TRPA1 Expression and NOS3-Mediated NO Release	Zhong et al. (2015)
	Schisantherin A		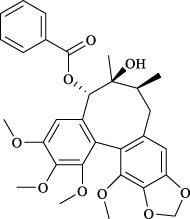		100 *μ*M	—		
	Deoxyschizandrin		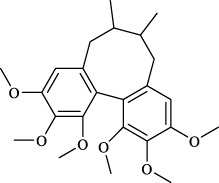		100 *μ*M	—		
	*γ*-schisandrin		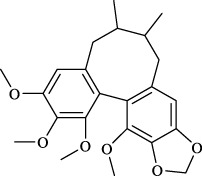		100 *μ*M	—		

## Commercialized Chinese herbal formulations and patent development status in asthma therapy

**FIGURE 4:**
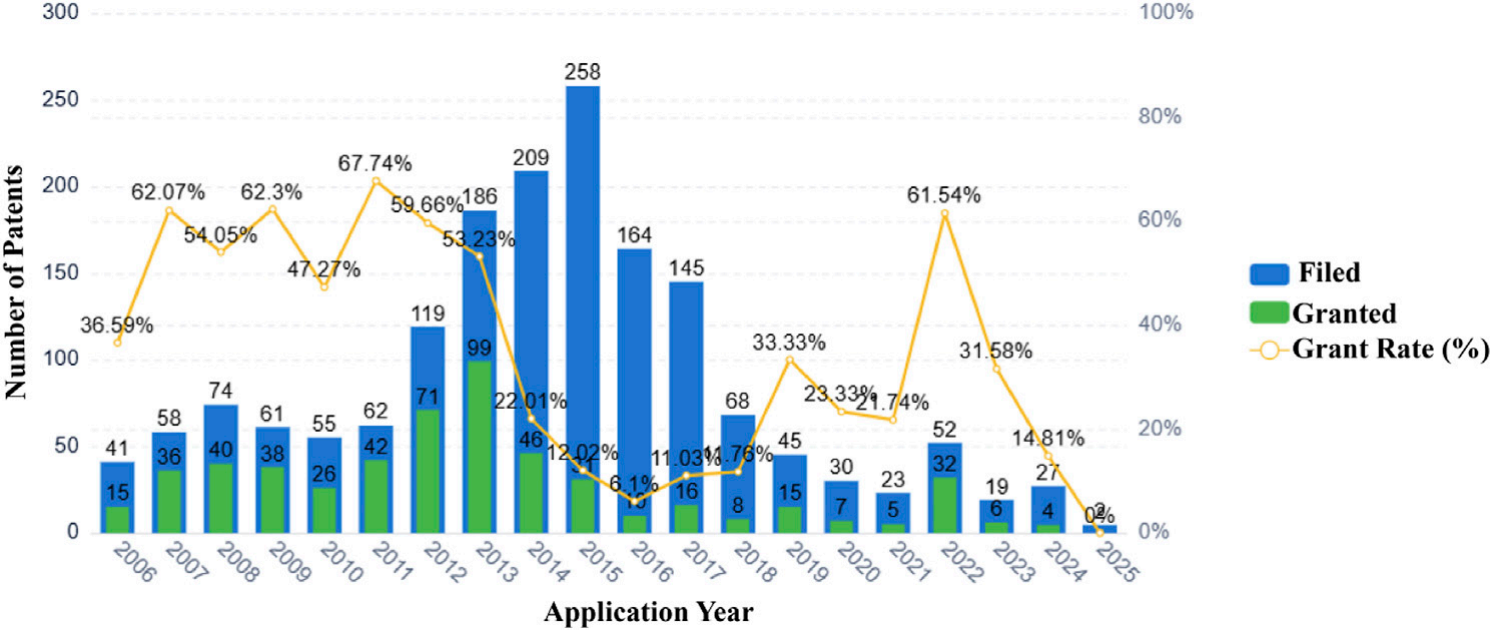
Patent applications and grants related to Chinese herbal medicines and asthma (2006–2025).

Currently, over 70 natural medicinal formulations extracted from CHM are approved for use in the Chinese market (see Supplementary Appendix 1). Despite these advances, significant challenges persist in translating CHM-related patents into clinically applied products. A systematic patent analysis using the keywords “Chinese herbal medicine” and “asthma” identified 1,835 relevant patent applications. Notably, analysis of patent filings over the past two decades reveals that only 32.2 percent of these applications were formally granted (Figure 4). Furthermore, only a small fraction of the granted patents progressed to the market implementation stage (see Supplementary Appendix 2).

Our analysis indicates that technological barriers and regulatory constraints are likely major factors contributing to the low patent conversion rate. The primary technological challenge stems from unclear active metabolites. Examination of extensive patent documentation reveals that most CHM patents describe only crude extracts, lacking definitive compound data, which renders them non-compliant with FDA/EMA registration requirements. Insufficient elucidation of mechanisms of action and a lack of robust clinical evidence further exacerbate this issue. Concurrently, regulatory deficiencies undermine patent value and impede investment. Firstly, a temporal misalignment exists between patent protection and drug registration. CHM formulations typically require several years to establish formulation stability and conduct pharmacodynamic validation before patent application. Combined with subsequent clinical trial durations, this significantly shortens their effective patent protection period compared to chemical drugs. This frequently results in new drugs reaching the market at or near patent expiration. Secondly, difficulties in infringement determination severely compromise patent value. Infringement assessment for chemical drugs relies on structural analysis of a single compound, whereas for CHM formulations, it necessitates concurrent proof of identical metabolites, consistent herbal ratios, and equivalent manufacturing processes.

## Conclusion and outlook

Asthma is one of the most common respiratory diseases encountered in clinical practice, characterized by a high incidence rate and a chronic course, imposing substantial physical, mental, and economic burdens on patients. Current clinical treatment strategies for asthma have certain limitations, underscoring the urgent need for the development of effective, targeted therapies with minimal side effects. CHM metabolites and their structural derivatives have emerged as an important source for new drug development due to their high safety, low toxicity, significant bioactivity, and diverse structures, thus making substantial contributions to disease treatment.

CHM demonstrates considerable advantages in the management of asthma. Firstly, with a historical trajectory extending over millennia, CHM has amassed a substantial body of clinical experience and traditional knowledge. This extensive repository of information offers valuable insights and guidance for the development of new pharmaceuticals, aiding in the identification of potentially therapeutic herbal metabolites and mitigating the element of randomness in research and development processes. Several CHM formulations, such as San’ao Decoction, Ma Xing Shi Gan Decoction, and She Gan Ma Huang Decoction, have been subjected to long-term clinical validation, thereby establishing a robust foundation for contemporary drug development. Secondly, the multi-target mechanism of CHM in the treatment of asthma offers significant advantages. Asthma is a complex disease characterized by multiple pathological processes, including airway inflammation, hyperresponsiveness, and immune dysregulation. CHM typically comprises various active metabolites that can concurrently act on multiple targets, thereby providing comprehensive therapeutic effects. This multi-target approach may offer superior benefits compared to single-target modern medications, facilitating a more holistic intervention in the asthmatic process and potentially reducing drug resistance and side effects associated with monotherapy. Thirdly, the diverse metabolites of CHM may exhibit synergistic interactions on specific biological targets. For example, in China, the combination of loquat leaf and Fritillaria has been traditionally employed in the management of respiratory diseases. As previously discussed, the alkaloids peiminine and peimine, derived from Fritillaria, substantially augment the TRPV1 inhibitory activity of ursolic acid found in loquat leaf. Notably, these alkaloids do not demonstrate direct TRPV1 inhibition when administered independently. Finally, CHM offers natural sources characterized by high safety profiles and potent biological activities, positioning them as potential reservoirs for innovative drug development. Through extensive biological evolution, natural products sourced from plants, animals, or minerals have exhibited remarkable biocompatibility with humans. Their high safety profiles and renewable nature have attracted considerable attention. Additionally, CHM encompasses numerous active metabolites and distinctive pharmacological effects, providing a rich foundation for innovation in contemporary asthma drug development. Comprehensive research and development of CHM may lead to the identification of new drug targets, novel active metabolites, and innovative therapeutic strategies, thereby advancing progress in asthma treatment. For example, the extraction of artemisinin from *Artemisia annua* L. [Asteraceae, Artemisiae Annuae Herba] has resulted in groundbreaking advancements in antimalarial drug development (Ma et al., 2020). Similarly, CHM may contain unique active metabolites with therapeutic potential for asthma that remain to be discovered.

The advancement of pharmaceuticals derived from CHM encounters several significant challenges. The intricate chemical composition and diversity of natural products inherent in CHM complicate the processes of screening, isolation, and structural characterization, rendering them both arduous and time-consuming. CHM, whether compound formulas or single botanical drug, comprise dozens or even hundreds of chemical metabolites, with pharmacokinetics and interactions of active metabolites often remaining ambiguous. This complexity poses substantial difficulties in aligning with contemporary pharmaceutical standards that demand ‘clear composition and well-defined mechanisms’. Moreover, existing literature predominantly concentrates on elucidating the mechanisms of active metabolites within preclinical animal models, while clinical data remain notably scarce.

Regarding the development of CHM targeting TRPV1/TRPA1, several challenges persist. Firstly, TRPV1/TRPA1 are widely distributed in the human body with complex regulatory mechanisms. Some natural drugs targeting these channels have limited clinical development due to side effects such as hyperthermia and thermal pain sensation impairment. Additionally, there are deficiencies in the research on the pharmacodynamics of CHM-modulating TRP channels for asthma treatment. While some studies confirm that certain CHM metabolites can alleviate asthma symptoms by activating TRPV1/TRPA1, drug development has primarily focused on TRPV1/TRPA1 inhibitors. It is well-known that certain exogenous or endogenous stimuli can trigger asthma responses by activating TRPV1/TRPA1. Therefore, how do some CHM metabolites provide therapeutic effects for asthma through TRPV1/TRPA1 activation without inducing asthma symptoms? It is due to receptor desensitization from neuropeptide depletion in sensory neurons, Ca^2+^ influx-driven receptor internalization after TRP channel activation, or receptor endocytosis and degradation after prolonged exposure to agonists. Whether these or other regulatory mechanisms exist requires further exploration. Additionally, whether the therapeutic effects of active metabolites from CHM or extracts are a direct result of interactions with TRPV1/TRPA1 or through other, as yet unexplained, mechanisms modulating TRP channels leading to indirect outcomes remains to be seen.

To address the aforementioned challenges, the following strategies may be implemented. Firstly, the integration of artificial intelligence (AI) and computational simulation technologies has the potential to significantly enhance the identification of active metabolites in CHM. For example, high-throughput virtual screening can efficiently identify candidate molecules with potential binding activity to TRPV1/TRPA1 from extensive libraries of CHM metabolites, thereby improving the efficiency of drug development and reducing associated costs (Zhang et al., 2025). Recently, a collaboration between Tasly and Huawei resulted in the creation of the “Digital Herb Intelligence Model”, which synthesizes over 90,000 formulas and three million data points on natural products, facilitating the intelligent generation of CHM formulations and predictions regarding their efficacy. Secondly, compared to the limitations of traditional methodologies for studying the mechanisms of CHM, multi-omics technologies (including genomics, transcriptomics, proteomics, and metabolomics) integrate multidimensional information at the molecular network level. This integration provides powerful tools for systematically elucidating the mechanisms by which CHM formulas and single botanical drugs regulate the TRPV1/TRPA1 channels. Furthermore, single-cell multi-omics technologies have advanced the research scale of precision medicine from tissues and organs to the single-cell level. These technologies reveal the dynamic regulatory effects of natural products on distinct cell types, thereby providing a key scientific foundation for CHM research. Thirdly, to address the potential side effects of TRPV1/TRPA1-targeting drugs, strategies such as optimizing drug formulations, employing localized targeted delivery systems to minimize systemic exposure, and implementing structural modifications of drug molecules can be employed. These approaches enhance the targeting specificity of candidate drugs, thereby improving their safety and efficacy while enhancing patient compliance. Lastly, randomized controlled trials (RCTs) are regarded as the gold standard for assessing the clinical efficacy and safety of pharmaceuticals, as they effectively minimize selection bias and confounding variables (Bothwell et al., 2016). Consequently, it is crucial to conduct additional RCTs on herbal active metabolites targeting TRPV1/TRPA1 to substantiate their therapeutic potential and safety profiles. A demonstrable relationship exists between the challenges of CHM drug development and the patent conversion difficulties outlined earlier. Implementing AI, multi-omics technologies, and RCTs may also offer potential to support the patent translation process.

In summary, although CHM holds great promise for developing new asthma treatments, overcoming its limitations and bringing these treatments to market will require substantial support and effort.
